# Validity, reliability, and bias between instrumented pedals and loadsol insoles during stationary cycling

**DOI:** 10.1371/journal.pone.0306274

**Published:** 2024-07-05

**Authors:** Walter Menke, Kaileigh Estler, Cary Springer, Songning Zhang

**Affiliations:** 1 Department of Kinesiology, Recreation, and Sport Studies, The University of Tennessee, Knoxville, TN, United States of America; 2 Office of Information Technology, Research Computing Support, The University of Tennessee, Knoxville, TN, United States of America; Ningbo University, CHINA

## Abstract

The purpose of this study was to evaluate the validity and reliability of the loadsol in measuring pedal reaction force (PRF) during stationary cycling as well as lower limb symmetry. Ten healthy participants performed bouts of cycling at 1kg, 2kg, and 3kg workloads (conditions) on a cycle ergometer. The ergometer was fitted with instrumented pedals and participants wore loadsol plantar pressure insoles. A 3 x 2 (Condition x Sensor Type) ANOVA was used to examine the differences in measured peak PRF, impulse, and symmetry indices. Root mean square error, intraclass correlation coefficients, and Passing-Bablok regressions were used to further assess reliability and validity. The loadsol demonstrated poor (< 0.5) to excellent (> 0.9) agreement as measured by intraclass correlation coefficients for impulse and peak PRF. Passing-Bablok regression revealed a systematic bias only when assessing all workloads together for impulse with no bias present when looking at individual workloads. The loadsol provides a consistent ability to measure PRF and symmetry when compared to a gold standard of instrumented pedals but exhibits an absolute underestimation of peak PRF. This study provides support that the loadsol can identify and track symmetry differences in stationary cycling which means there is possible usage for clinical scenarios and interventions in populations with bilateral asymmetries such as individuals with knee replacements, limb length discrepancies, diabetes, or neurological conditions. Further investigation of bias should be conducted in longer cycling sessions to ensure that the loadsol system is able to maintain accuracy during extended use.

## Introduction

The availability of biomechanics research equipment that reduces the financial cost, size, and operator knowledge has increased substantially in the past decade. New devices and algorithms have allowed for the development of novel, advanced techniques for measuring biomechanical variables in ways unavailable to traditional equipment and methods [1–4]. One such system is the loadsol force measuring insole produced by novel®. The loadsol is a wireless, Bluetooth® insole used to measure in-shoe pressure in the form of vertical ground reaction force (GRF_z_) via plantar pressure sensors. Systems such as the pedar insole system are considered the gold standard for measurement of plantar pressure, but the cost and specialized knowledge to operate present a barrier to entry for deployment in clinical settings. The pedar is a higher resolution plantar pressure system that has been used to evaluate the effects of orthoses in cycling, the relationship between plantar pressure and ulcers in diabetic populations, as well as shoe stiffness in diabetic footwear [5–7]. Additionally, the pedar has been shown to be more valid and reliable than other, similar systems such as Medilogic and Tekscan as well as less biased in center of pressure measurements compared to the loadsol [8, 9]. Since the pedar system consists of 99 capacitive sensors, it allows for a finer grained analysis of the whole foot compared to the loadsol. The loadsol system provides a simpler solution for measuring GRFz for wider applications as a result of the cost, ease of calibration, and data collection simplicity. The loadsol is able to record GRFz in three distinct regions: the heel, lateral forefoot, and medial forefoot as opposed to the many more regions of the pedar system.

Previous research has assessed the validity and reliability of the loadsol for quantifying GRF_z_ during tasks such as running, walking, hopping, and landing, as well as applications for evaluating asymmetry in clinical populations and return to sport assessments [10–16]. Evaluation of lower limb kinetic symmetry is of interest to clinicians that work with populations such as individuals with total knee replacements or athletic populations [17]. In these cases, GRFz may be used as a proxy for joint loading and loading symmetry is desirable to, for example, minimize potential wear on an implant or cartilage [18–20]. Burns [10] reported good to excellent agreement with intraclass correlation coefficients of 0.96 for control participant hopping on a force plate and 0.88–0.96 for walking and running on an treadmill. Loiret [13] quantified gait asymmetry in transfemoral amputees by comparing the loadsol metrics with force plates. The findings concluded that the insoles were easily usable in clinical settings and had correlation coefficients ranging from 0.91–0.95, demonstrating a high level of agreement. Seiberl [11] utilized the loadsol to examine loading symmetry in patients with anterior cruciate ligament reconstruction during hop testing and concluded the sensors had a high accuracy with mean biases ranging from 0.6 to 3.4% of the variables of interest such as ground contact time, impulse, and peak force.

These various bodies of work provide support for the efficacy of the loadsol as a valid and reliable measurement device for GRFz as well as its viability for clinical settings. However, the loadsol has not been evaluated in a stationary cycling task, which is often used in the rehabilitation and symptom management for those with lower limb osteoarthritis (OA) or total knee arthroplasty (TKA) surgery recovery [21, 22]. Cycling tasks have an inherent level of intra- and inter-subject variability for healthy populations as well as knee OA populations, which suggests that the ability of the loadsol to quantify of this variability should be investigated [23, 24]. Considering that clinical rehabilitation spaces infrequently have access to expensive and sophisticated biomechanical equipment for measuring kinetics such as instrumented pedals, a more affordable and mobile device such as the loadsol may improve its adoption in clinical settings. As a result, the loadsol should be evaluated for its validity and reliability during cycling tasks as well as the degree of accuracy in detecting lower limb kinetic symmetry to be a more robust tool for clinicians.

Therefore, the purpose of this study was to validate and quantify the loadsol’s ability to objectively measure peak PRFz and its impulse, assess the presence of measurement bias, and determine the ability to properly identify lower limb kinetic symmetry when compared to those measured by pedals. It was hypothesized that the loadsol would have good agreement with the pedals across all metrics and not display systematic or proportional biases, indicating that the loadsol is a valid device for measuring PRFz in cycling tasks.

## Methods

Ten healthy college aged students (Male = 5) were recruited to participate in the study (age = 22±4 years, height = 1.70±0.1 m, mass = 75.6±12.6 kg, BMI = 26±2.9). The recruitment period for this study started on 07/02/2022 and ended on 21/10/2022. Inclusion criteria for this study consisted of no previous lower extremity surgery, no lower extremity injury within the last 6 months, between the ages of 18 and 35, ability to ride a stationary bicycle without external aid, and participating in non-cycling moderate-to-vigorous physical activity at least 3 days per week. Cycling experience per week was limited to a maximum of 3 hours. Participants were only included in the study if they answered “no” to every question on the PARQ+, to ensure they are healthy enough to exercise. To obtain an α of 0.05, a β of 0.80 and an effect size of 0.75, a minimum of 10 participants total were needed as assessed through a power analysis conducted in GPower 3.1 [25]. Frontal plane joint moments from previous literature were used as inputs into GPower [26]. All participants signed an informed consent document approved by the Institutional Review Board at the University of Tennessee.

A motion capture system (240 Hz, Vicon Motion Analysis Inc., UK) with 12 cameras was used to obtain the three-dimensional (3D) kinematics during the test. Retroreflective anatomical markers were placed on bony landmarks of interest while clusters of markers affixed to semi-rigid thermoplastic shells were placed on the trunk, pelvis, and bilateral thighs, shanks, and heels of each participant to track segmental motion. Additional markers were used to define the pedals and track motions of each bike pedal and crank arm.

A pair of 3-region (heel, medial forefoot, lateral forefoot) wireless pressure sensor insoles (100Hz, loadsol, novel Munich, Germany) were inserted into both shoes to measure PRF_Z_ during cycling trials. The loadsol data were recorded via the loadsol mobile app, loadsol-s, on an iPad and saved to a laboratory specific cloud drive. The loadsols were calibrated using the manufacturer process that includes having the participant stand entirely on each foot to zero the sensor of the lifted foot. A customized set of instrumented pedals (Pedal) with two 3D force sensors (1200 Hz, Type 9027C, Kistler, Switzerland) coupled with two industrial charge amplifiers (Type 5073A and 5072A, Kistler, Switzerland) were used to measure 3D PRFs on the ergometer PRFs, loadsol and marker data collection were conducted simultaneously using the *loadsync* device (novel, Munich, Germany), and Vicon system and Nexus software suite. The *loadsync* synchronizes data collection of 3D kinematic, Pedal data, and loadsol data. The instrumented pedals were hardware and software zeroed before each trial, but did not require any specific calibration before testing. A Monark Ergometer (Model 818E, Monark, Varberg, Sweden) was used for the stationary cycling testing.

Participants attended one testing session. Prior to the test session, participants completed an informed consent form, PARQ+, and basic information, demographic, and physical activity sheets. Prior to testing, participants were guided through a standard procedure to ensure proper bike fit. The saddle height for each participant was set corresponding to a knee flexion angle of 25–30 degrees. The angle of flexion was measured via handheld goniometer with the Pedal positioned at the bottom dead center position [27]. The handlebar position was adjusted to ensure a trunk angle of 90 degrees. The seat fore-aft position was adjusted to ensure the patella is vertically aligned with the pedal spindle with the pedal positioned at the bottom dead center; measured using a plumb bob [26, 28]. For the testing session, participants completed a three-minute cycle ergometer warm-up at a self-selected cadence with a workload of 0.5kg. The loadsol*® devices were zeroed while standing according to the loadsol iPad application instructions before testing began*.

Participants were tested in three stationary cycling conditions, which consisted of cycling in three workload conditions of 1.0 kg, 2.0 kg, and 3.0 kg at 80 revolutions per minute (RPM). The testing order of workload conditions was randomized for each subject. The participants wore standardized lab running shoes (Air Pegasus, Nike), which were strapped onto the Pedal with a toe-cage to the participant’s comfort. The participant cycled for 2 minutes under each test condition and data were collected during the last 10 seconds. Between the cycling conditions, the participant was asked to step off the ergometer for the researchers to zero the Pedals before proceeding with the next condition. Cadence was provided visually with a number on the ergometer display, and researchers verbally reminded participants of the cadence requirement if they deviated from the goal cadence for more than a few pedal revolutions. Participants were asked to maintain 80 RPM as best as they could, with an error range of ± 2 RPM. Participants were given a minimum of 2 minutes of rest between testing conditions, or as long as needed.

Visual3D (C-Motion, Inc., Germantown, MD, USA) 3D biomechanical analysis software suite was used to compute 3D PRFs. Customized computer programs (Microsoft Visual BASIC, 6.0, MATLAB R2022b) were used to compute and determine critical events of the computed variables from Visual3D outputs and organize data. PRF data were filtered using a fourth order zero-lag Butterworth lowpass filter with a cutoff frequency of 6 Hz [26, 28]. PRF data were not normalized to participants’ body weight due to the partial support of bodyweight by the saddle [29]. Loadsol *data were up-sampled to 1200 Hz to match the sampling rate of the Pedals and to allow root mean squared error (RMSE) to be calculated between the two devices*. *Vertical force from the loadsol wholefoot (loadsol) was used for all comparisons to the Pedals*. *Bilateral RMSEs were calculated separately to compare the left loadsol force taken from the loadsol with the left PRF*_Z_ *and similarly for the right side*. *The kinetic data from the loadsol were not filtered due to the minimal noise seen in raw loadsol data in cycling*. *Deciding not to filter the loadsol data also preserved the peaks of the plantar pressure*. *The data assessed consisted of the entire 10 second trial period and were not broken down into individual crank cycles*. *This choice was made because most clinics will not have the capability to synchronize their loadsol data to the true pedal and crank coordinate system during cycling*. *As a result*, *we found it externally valid to use the entire 10 second period as a trial*. *Further*, *during a 10 second period at 80 RPM*, *there will be approximately 13 cycles present in the data*, *which is sufficient for assessing device measurement properties*. Symmetry index (SI) was calculated to compare the left and right-side PRF_Z_ from the respective Pedal (SI_pedal_) and loadsol (SI_loadsol_).


$$Symmetry\;Index=(\frac{Right\;side\;variable-Left\;side\;variable}{Right\;side\;variable})*100$$

*A positive output value indicates the right-side variable being larger*, *and a negative value indicates the left side variable being larger*. *The values obtained for Pedal vertical reaction force (PRF*_z-_*pedal) and loadsol force (PRF*_z-loadsol_*) were compared for agreement between the two devices*. *Vertical PRF* impulse and peak force for the entire 10 second trial were computed from measurements derived from both devices separately.

MATLAB (2022b, MathWorks, Natick, Massachusetts, USA) and SPSS (v29, IBM, Armonk, New York, USA) were used to produce graphical results and perform statistical tests, respectively. Two separate 3x2 (Condition x Sensor) repeated measures analysis of variance (ANOVA) were performed with Bonferroni corrections to assess differences in the SIs by condition as described above. One-way ANOVAs were used to compare PRF_z_ and loadsol as well as the to compare bilateral RMSE data. An *α* level of 0.05 was set a priori. Intraclass correlation coefficients (ICCs) were computed for impulse and peak reaction forces to compare the agreement of these variables between the two devices. ICCs were computed using a Two-Way Mixed Effects model for Consistency with a 95% confidence interval in SPSS. ICCs with values of less than 0.5, between 0.5 and 0.75, between 0.75 and 0.90 or greater than 0.9 were classified as poor, moderate, good, and excellent reliability, respectively [30]. Impulse and peak reaction forces were also imported into MedCalc (Ostend, Belgium) for Passing-Bablok regressions to evaluate the presence of systematic and proportional bias [31]. The evaluation for an incidence of systematic bias was assessed by the presence of 0 within the intercept 95% confidence interval (CI) while proportional bias was assessed by the presence of 1 within the slope 95% CI.

## Results

No significant effects were found for SI comparisons between devices or workloads for the vertical comparison between Pedal (SI_pedal_) and loadsol (SI_loadsol_) (Table 1). SIs between the left and right loadsol were -25.5%, -4.23% and -6.47% for the 1kg, 2kg and 3kg workloads, respectively. SIs between the PRF_z_ of left and right sides were -3.09%, -1.28% and -2.5% for the 1kg, 2kg, and 3kg workloads, respectively.

**Table 1:** Vertical SI values (%) by condition and sensor type: mean ± standard deviation.

	*1kg*	*2kg*	*3kg*	**P-Values** *Condition Sensor Interaction* 0.211 0.314 0.284
SI_loadsol_	-25.50± 54.74	-4.23± 32.16	-6.47± 16.85	
	*1kg*	*2kg*	*3kg*	
SI_pedal_	-3.09± 12.19	-1.28± 5.51	-2.50± 6.46	

No significant difference effects were found for RMSE values between the loadsol and Pedals of left and right sides (Table 2). Within the left side when comparing the RMSE (p = 0.263) between sensor types, there was a 46.6N, 52.4N and 64.9N mean difference for the 1kg, 2kg and 3kg conditions, respectively. Within the right side RMSE (p = 0.565), there was a 50.4N, 49.1N and 57.8N mean difference for the 1kg, 2kg and 3kg conditions, respectively.

**Table 2:** RMSE between PRF_z-pedal_ and PRF_z-loadsol_ for Right and Left Sides (N): mean ± standard deviation.

	Condition			P Value
	*1kg*	*2kg*	*3kg*	
Left	46.56±20.88	52.36±30.10	64.85±28.53	0.263
Right	50.38±28.90	49.07±23.87	57.80±29.14	0.565

**Fig 1:**
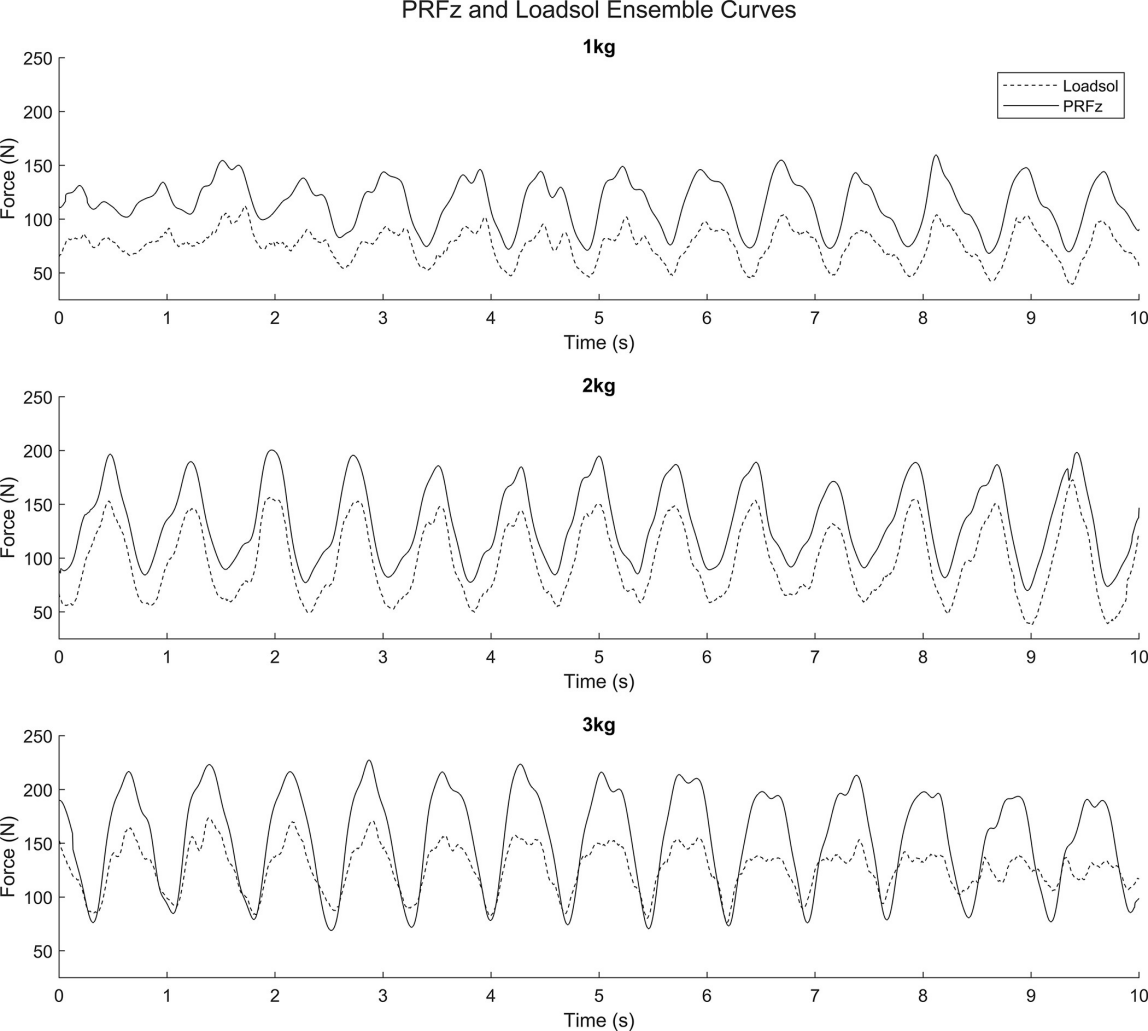
PRF_z-pedal_ and PRF_z-loadsol_ ensemble curves for the right pedal and foot only across all workloads.

The ensemble curves for all workloads of the right side are provided in Fig 1. Peak vertical PRFs ICCs ranged from 0.454 to 0.954 and the vertical PRF impulse ICCs ranged from 0.325 to 0.850 (Table 3). The loadsol did not show any systematic or proportional bias for any of the tested regressions for peak PRFs (Table 4). The loadsol exhibited systematic bias only when assessing all samples across all conditions for the impulse measurements with no bias seen for individual workloads.

**Table 3:** Average measures ICC values for PRF_z_ and total impulse between loadsol and instrumented pedals.

	Right Side			Left Side		
	*1kg*	*2kg*	*3kg*	*1kg*	*2kg*	*3kg*
Peak PRF_z_	0.954	0.713	0.868	0.788	0.454	0.892
Impulse	0.325	0.663	0.754	0.549	0.531	0.850

**Table 4:** Passing-Bablok regression results and bold text indicates presence of respective bias.

	**Peak PRFz**			
	*All* (n = 30)	*1kg* (n = 10)	*2kg* (n = 10)	*3kg* (n = 10)
Systematic Intercept	-29.95	-16.31	-37.84	157.43
95% CI	-115.37, 19.87	-170.15, 71.60	-898.80, 210.07	-84.94, 290.38
Proportional Slope	0.97	0.91	0.99	0.47
95% CI	0.80, 1.22	0.53, 1.24	0.26, 3.31	0.13, 1.13
	**Impulse**			
	*All* (n = 30)	*1kg* (n = 10)	*2kg* (n = 10)	*3kg* (n = 10)
Systematic Intercept	**-793.60**	-1054.30	-666.09	-532.75
95% CI	**-2117.72, -90.15**	-6154.31, 635.73	-7179.76, 788.24	-4213.85, 754.48
Proportional Slope	1.37	1.53	1.24	1.21
95% CI	0.88, 2.23	0.21, 5.2608	0.30, 6.00	0.35, 3.60

**Fig 2:**
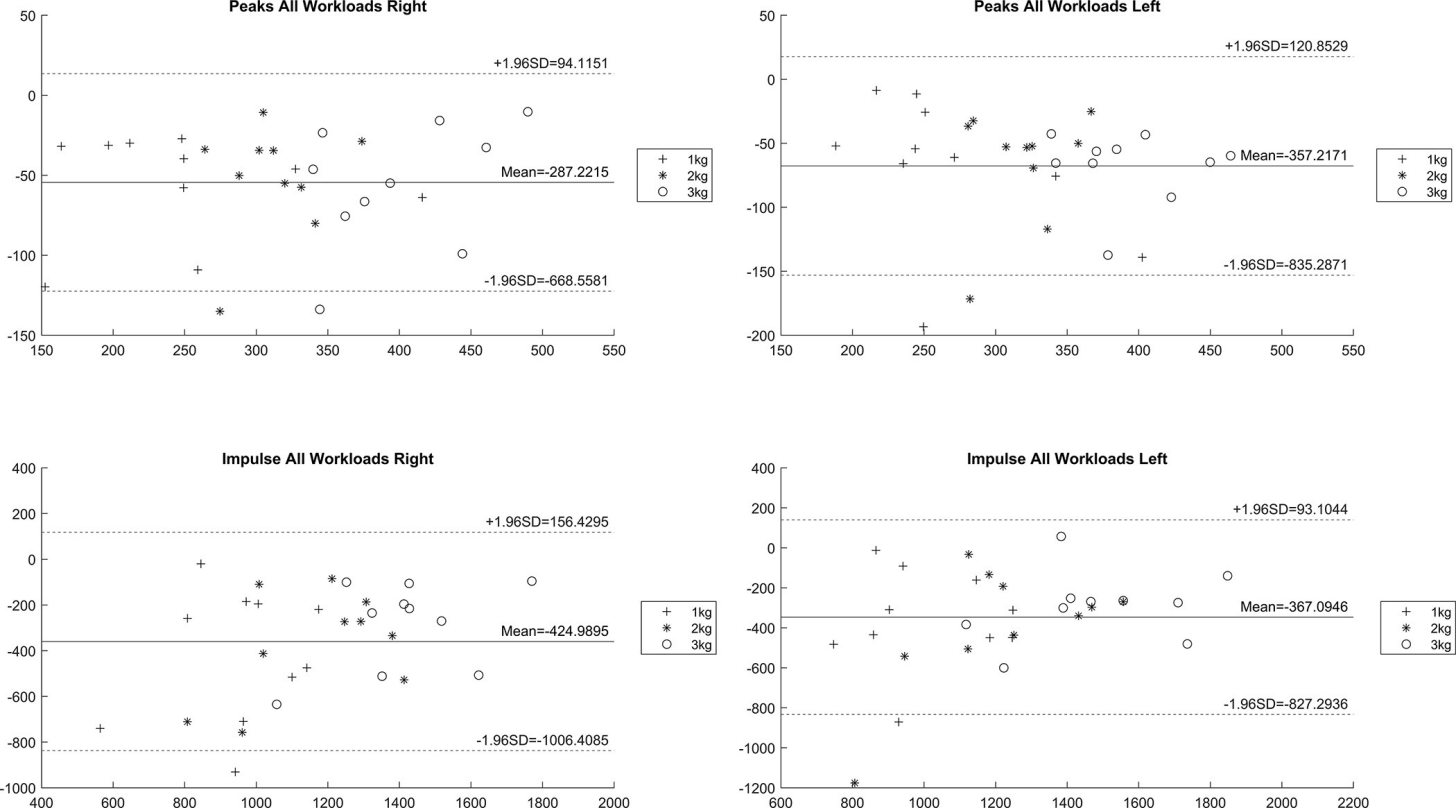
Bland Altman plots for peak left and right PRFz and impulses for all workload conditions.

Fig 2 provides Bland-Altman limits of agreement plots for peak and impulse values for all workloads, 1kg to 3kg, for the right and left pedals.

## Discussion

The purpose of this study was to assess the validity and reliability of the loadsol for usage in stationary cycling and lower limb symmetry assessment applications. The hypothesis was not supported regarding validity as the loadsol exhibited poor to excellent agreements across the evaluated variables, but did exhibit consistent reliability.

SI comparisons for PRF_z_ showed no significant effect for condition, sensor type or their interaction which indicates that the loadsol can perform similarly to the gold standard of instrumented pedals. The value of all SIs were negative indicating that the participants seemed to favor their left foot slightly. The absolute value of the SI_loadsol_ offset from zero were generally small which indicates that the loadsol is sensitive enough to identify asymmetric patterns of external load in healthy populations and that can likely be utilized for clinical populations with a higher degree of asymmetry.

All RMSE values showed that the Pedals consistently measured higher values than the loadsol with a range of approximately 45–65 Newtons; but nothing of statistical significance was found. In this study, the Pedal values are considered as the true PRF_z_ value and therefore the loadsol has consistently underestimated the PRF_z_. This trend can be also visualized in the ensemble curves (Fig 1) where it can be seen that the values are underestimated, but the timing and patterns match quite well with the PRF_z-pedal_. Similar trends of underestimation were also seen in previous loadsol studies [11, 14]. Peebles [14] evaluated the loadsol at 100 Hz and 200 Hz during landing conditions in comparison to force plate measures and still found significant biases in peak impact force that increased with increasing load even with the 200Hz loadsol model. Lower limb symmetry of dependent variables between lower limbs, was also assessed by Peebles [14] and symmetry measures did agree on one day of testing, but the repeatability of symmetry testing ICCs ranged from 0.004 to 0.880. Seiberl [11] discovered a 3.4% underestimation of peak force by the loadsol during running conditions. Previous literature and our results raise some concerns about underestimation of peak force of the loadsol during high and low force activities such as landing and cycling. In an application where timing is of interest and magnitude is not as much of a concern, the loadsol may be a valid utility. However, when the activity is highly dynamic the temporal accuracy may be suspect due to the lower sampling frequency compared to an embedded force plate [11]. While cycling is a repetitive, decreased force activity compared to hopping and landing the accuracy of timing may still be relevant for populations with neurological conditions causing muscle dysfunction during cycling such as cerebral palsy [32].This finding suggests the possibility of systematic bias within the loadsol regarding underestimation, however no systematic bias was found statistically for peak reaction force or impulse values when assessing individual workloads in the Passing-Bablok regression. Systematic bias only occurred when all conditions were aggregated for an *n* of 30 for the impulse metric. It should be noted that the systematic CIs relating to impulse for the separate workloads were incredibly large and while not statistically significant, caution should be taken when interpreting force-time-based variables such as impulse derived from loadsol measurement. Considering a consistent underestimation of peak forces by the loadsol in stationary cycling, if a trial contains a multitude of changing peak values over a long period of time our results suggest the impulse derived will not be highly accurate. However, the effects of trial length may not be present when assessing individual crank cycles of 360֯, for example. No proportional bias was detected for peak PRF_z_ or impulse and provides support for the conclusion that the loadsol offset does not become worse as workload increased, which is contrary to findings by Peebles [14]. ICC values ranged from poor to excellent for peak reaction force and impulse measurements across the test conditions. The ICCs for peak PRFz for all three workloads ranged from 0.713 to 0.954 for the right side and from 0.454 to 0.892 the left side. The disparity between values for each side highlights a potential issue with the study design or experimental procedure. In addition, the ICC values for impulse were lower than the peaks for all but one condition. The ICC findings for impulse support the results from the Passing-Bablok in that temporal metrics taken from the loadsol should be assessed carefully and may not be suited for certain dynamic tasks.

The varied findings from previous literature may not only stem from the specific type of activity that is being investigated, but also in unreported calibration and zeroing procedures. In this study, the loadsols were zeroed before the first trial only. It is possible that other studies zeroed the loadsols between trials. In the absence of zeroing the loadsols on a per trial basis, the possibility of baseline drift cannot be discounted. However, given that these results do not suggest systematic or proportional bias for each individual workload, it is unlikely that sensor drift plays a role as a confounding variable. Considering that clinical applications generally do not consist of high intensity or endurance length cycling sessions, these results suggest that the reliability of the loadsol system is acceptable for clinicians interested in shorter duration cycling training sessions. The presence of systematic bias would be problematic for clinicians in cases where over- or under-representing plantar force and could cause different clinical conclusions. For example, interventions can be affected by systematic bias in a pair of loadsols where clinicians may be led to believe that their diabetic patients are applying greater pressure during cycling with custom foot orthoses than they truly are. Our results suggest that there is no reason to believe the loadsol insoles are not able to adequately measure plantar pressure symmetry, which is beneficial for clinical situations and populations where consistent evaluation of each limb is of interest.

Limitations of methodology in this study may have altered the significance of results. While the power analysis suggested a sample size of 10, a sample of 10 is still objectively small to draw concrete conclusions from. The loadsol has previously been criticized as needing an improved calibration protocol which may have affected this study’s results (Loiret 2019). It is possible that the toe cages were tightened differently for a given side or between participants and the external pressure on the metatarsals translated to greater internal foot pressure as measured by the loadsol. loadsol fit for this study was not strictly controlled and the pairs assigned to a participant matched their self-reported shoe size which may have resulted in the foot surface area coverage of the loadsol being poor.

## Conclusions

The loadsol appears to provide a reliable and consistent platform to assess PRF and evaluate useful clinical metrics in accordance with a gold standard when assessing RMSE outcomes. However, the ICC results revealed that there can be poor agreement between limbs and workload conditions in stationary cycling and the validity of peak metrics are in question. Passing-Bablok regressions provide support for the view that the loadsol has little to no systematic and proportional biases. As a result, it can be concluded that, the loadsol will perform as a consistent device for measuring PRF and calculating lower limb symmetry during stationary cycling. The implications of this work for clinicians are that the loadsol can provide a consistent and convenient method of measuring GRFz and tracking kinetic asymmetry throughout rehabilitation progress in stationary cycling. Clinicians concerned with populations such as total knee replacement recipients, diabetics, or those with limb length discrepancies can also track GRFz with increases in resistance during stationary cycling.
